# Boy–Girl Differences in Pictorial Verbal Learning in Students Aged 8–12 Years and the Influence of Parental Education

**DOI:** 10.3389/fpsyg.2018.01380

**Published:** 2018-08-08

**Authors:** Marleen A. J. van Tetering, Renate H. M. de Groot, Jelle Jolles

**Affiliations:** ^1^Centre for Brain and Learning, Faculty of Behavioural and Movement Sciences, Vrije Universiteit Amsterdam, Amsterdam, Netherlands; ^2^Welten Institute, Research Centre for Learning, Teaching and Technology, Open University of the Netherlands, Heerlen, Netherlands; ^3^NUTRIM School of Nutrition and Translational Research in Metabolism, Maastricht University, Maastricht, Netherlands

**Keywords:** intentional learning, school achievement, schoolchildren, individual differences, sex differences, parental education

## Abstract

This large-scale cross-sectional study of schoolchildren aged 8–12 years (*N* = 152) evaluates two factors which potentially determine individual differences in intentional learning: the child’s sex and parental education. Intentional learning was assessed with a newly constructed Pictorial Verbal Learning Task (PVLT). This task presents line drawings of concrete objects as to-be-remembered information instead of written or auditory presented words. The PVLT has the advantage that performance is not confounded by individual differences in reading or hearing abilities. Results revealed clear sex differences in performance: Girls outperformed boys. Parental education also contributed to individual differences in performance since children of higher educated parents outperformed children of lower educated parents. The results therefore suggest that both sex and parental education could be potent contributors to individual differences in learning performance at school. The findings more specifically imply that children of less educated parents and boys need additional guidance and support in intentional learning when new information and procedures are presented for the first time.

## Introduction

Intentional learning is of key importance for the acquisition of new information and for academic performance. The process of intentional learning can typically be described as having the purpose of learning information and committing it to one’s memory (Thomas and Rohwer, 1986; Lezak et al., 2012; Hampshire et al., 2016). This is different from incidental learning, which is the accidental learning of information while actually concentrating on other information (Thomas and Rohwer, 1986; Lezak et al., 2012; Hampshire et al., 2016; Ahmed, 2017; Kontaxopoulou et al., 2017). The ability of intentional learning typically improves with experience and with age (Meijer et al., 2006, 2007; Meijs et al., 2013, 2016; Blachstein and Vakil, 2016). It appears that there are major individual differences in the pace at which intentional learning develops at the end of childhood and the beginning of adolescence (Meijer et al., 2007; Jolles, 2016; Meijs et al., 2016; Juraska and Willing, 2017).

Individual differences in intentional learning performance may contribute to individual variations in the ability to acquire and consolidate new information at school, as well as in learning motivation and school achievement. Examples of intentional learning at school is when students have to learn lists of words that belong to a new language and when they have to learn lists of conjugations of verbs. Another example of intentional learning at school is when new mathematical rules and multiplication tables need to be learned. Students with poor intentional learning ability may experience profound difficulties with learning lists of new words, conjugations of verbs as well as with learning new mathematical rules and lists of tables. This may negatively affect their language and mathematic performance as well as their learning motivation (Lawrence et al., 2015). It is therefore of importance to find out whether students are characterized by individual differences in intentional learning performance, and whether there are external factors that contribute to these individual differences. Identification of external factors could allow early detection of those individuals who are at risk of poor intentional learning performance, and thus also for problems with learning at school. It could also enable the formulation of intervention programs that aim to stimulate the development of intentional learning ability (Dekker and Jolles, 2015). The present large-scale study aims to evaluate the notion that there are at least two risk factors for inferior intentional learning performance in students aged 8–12 years, namely male sex and lower parental education.

The notion that sex and level of parental education (LPE) may contribute to individual differences in intentional learning is, in part, based upon findings that boys and girls, and children of higher and lower LPE families differ in their school achievement. The average school achievement of girls is higher than that of boys in the age period between 8 and 12 years (see Van der Elst et al., 2012; Voyer and Voyer, 2014; Organisation for Economic Cooperation and Development [OECD], 2015; Stoet and Geary, 2015; Williams et al., 2015; Kontaxopoulou et al., 2017). Likewise, there is a rapidly growing volume of literature indicating that the average school achievement of children of lower educated parents are significantly lower than those of their peers who have higher educated parents (e.g., Davis-Kean, 2005; Rindermann and Baumeister, 2015; Inspection of Education the Netherlands, 2015; Chiu et al., 2016). The sex and LPE differences in school achievement suggest that boys and girls, and children of higher and lower LPE families could differ in various cognitive abilities that are important for learning and performance at school.

Previous studies have reported on sex differences in various cognitive tasks. For instance, there is ample scientific evidence that girls outperform boys in verbal fluency tasks and tasks that evaluate inhibitory control (e.g., Hyde and Linn, 1988; Berlin and Bohlin, 2002; Dekker et al., 2013b). Other studies showed that boys outperform girls on spatial ability tasks (e.g., Voyer et al., 1995; Hoyek et al., 2011; Miller and Halpern, 2014). These findings indicate that sex differences on various cognitive tasks do exist. The notion that sex is a determinant of individual differences in performance on cognitive tasks is further substantiated by findings of neuroimaging studies. These studies reported on sex differences in the maturation of brain areas and networks (Lenroot and Giedd, 2010, 2011; Miller and Halpern, 2014; Hampshire et al., 2016; Juraska and Willing, 2017). It appears that total cerebral volume and gray matter volume peak at a later age in boys (14.5 years) than in girls (10.5 years) (Giedd, 2008). These sex differences in brain maturation and on cognitive tasks are also discussed in the authoritative review ‘The new science of cognitive sex differences’ of Miller and Halpern (2014). In their review, Miller and Halpern (2014) reported on studies which showed that cognitive sex differences are changing over time: They are decreasing on some tasks whereas remaining stable or increasing on other tasks. They therefore concluded that it is needed to reinvestigate sex differences on various cognitive functions, including intentional learning ability.

With respect to differences between children of higher and lower LPE families on cognitive abilities, previous studies reported that children of higher educated parents tend to have superior verbal abilities, a larger vocabulary, and more rapid language development than children growing up in lower LPE families (Ganzach, 2000; Hoff, 2003; Carr and Pike, 2012; Kautz et al., 2014). Differences between children of higher and lower LPE families have also been demonstrated in problem-solving behavior and attention (Hurks et al., 2006; Meijs et al., 2009), and they have been reported by teachers. Teachers observed that planning and initiative taking behaviors were higher for children with higher LPE than for children with lower LPE at the ages of 8–12 years (van Tetering and Jolles, 2017). The better cognitive performance of children from higher LPE families has been attributed to genetic factors as well to difference in environmental factors such as a more inspiring and intellectually stimulating atmosphere in which children grow up (see van Soelen et al., 2009; Sameroff, 2010; Trzaskowski et al., 2014). In addition to teacher observations, neuroimaging studies reported on associations between parental education and total brain surface area (Noble et al., 2015). These findings indicated that any increase in parental education – for example, an extra year of high school or at college – was associated with an increase in surface area over the course of childhood and adolescence (Noble et al., 2015). Taking the findings of all of these studies together it is suggested that sex and LPE are relevant factors contributing to individual differences in cognitive development. Both factors may, therefore, also contribute to individual differences in intentional learning performance.

Sex differences and differences between children of higher and lower LPE families in intentional learning ability have been reported by previous studies using classical verbal learning tests, such as the Rey Auditory Verbal Learning Task (AVLT, Rey, 1964) and the California Verbal Learning Test (CVLT, Eric, 1987; e.g., Kramer et al., 1997; McDermott et al., 2001; Vakil et al., 2010). Both typical intentional learning tasks consist of a learning phase. In this phase, the participants are presented with to-be-remembered information such as spoken or written words. Before this learning phase, participants are told to remember as many pieces of information as possible because they will be requested to recall the information afterward (Ahmed, 2017). These kinds of tasks are analogous to various aspects of learning at school and in daily life, such as when learning a list of facts, rules, or words. Both the AVLT and the CVLT are frequently used for the evaluation of intentional learning in clinical settings (Rey, 1964; Van der Elst et al., 2005; Lezak et al., 2012; Correia and Osorio, 2013; Williams et al., 2015; Emami et al., 2017; Nunez et al., 2017).

Sex differences and differences between children of higher and lower LPE families in performance on classical intentional learning tasks, however, give no clear answer to the question whether there are sex and LPE differences in intentional learning performance. For instance, a problem with CVLT is that it offers to-be-learned information in written words (e.g., CVLT; Van der Elst et al., 2005; Lezak et al., 2012; Correia and Osorio, 2013). Performance on this task therefore relies heavily on reading ability. This is an important notion when studying intentional learning performance in children, as it has repeatedly been shown that girls have superior reading abilities compared to boys (e.g., Hyde and Linn, 1988; Hoff-Ginsberg, 1991; Hoff, 2003; Miller and Halpern, 2014). Superior performance of girls in classical verbal learning tasks may, therefore, represent their superior reading abilities rather than superior performance in intentional learning. Likewise, accumulating evidence shows that children who grow up in higher LPE families have superior reading abilities compared to children growing up in lower LPE families (e.g., Hoff-Ginsberg, 1991; Hoff et al., 2002; Hoff, 2003). Previous studies showed that children from lower LPE families lag behind in their reading development because they have gained less experiences with reading books, and the language and verbal communication practiced in their family is less complex than in higher LPE families (Hoff-Ginsberg, 1991; Hoff et al., 2002; Hoff, 2003). Lower performance of children of lower LPE families on the CVLT may therefore represent their poorer reading abilities rather than poorer performance in intentional learning. Moreover, other classical verbal learning tasks – such as the AVLT (Rey, 1964) – cannot be used by children that have hearing difficulties. In order to investigate sex differences and the importance of LPE to intentional learning without major confounding by pre-existing differences in reading and hearing abilities, the present study introduces a newly developed task; the Pictorial Verbal Learning Task (PVLT).

The uniqueness of the PVLT comes from the modality in which to-be-learned information is offered, which is pictorially. By showing pictures we controlled for individual differences in receptive language processing because children do not need to read or listen to words. The task does require children to understand the pictures conceptually and translate them into expressive language (spoken or written language). The task used in this study therefore has the advantage compared to classical verbal learning tasks of being less sensitive to pre-existing individual differences in reading and hearing abilities (i.e., receptive language), which can influence learning performance. Another advantage of the PVLT is that it assesses intentional learning as important for learning at school and in daily life where information is often presented pictorially. Performance on the PVLT is analogous to that on the AVLT and the CVLT, and reflects intentional learning since the subject gets the explicit instruction to remember as many pictures as possible. After each trial, they are asked to recall as many pictures as they have remembered. An important note with regard to the use of pictures as stimuli is that it has repeatedly been shown that visuospatial abilities of boys are superior to those of girls (e.g., Voyer et al., 1995; Hoyek et al., 2011; Miller and Halpern, 2014). A pictorial task could, therefore, be to the advantage of boys because visual perception and recognition are more important in such a pictorial task than word recognition. If girls outperform boys on the PVLT, it would thus give even stronger support to our hypothesis that there are sex differences in intentional learning.

In order to investigate sex and LPE differences on the PVLT, a large-scale cross-sectional study was conducted with children aged 8–12 years. Specifically, this study investigated (1a) whether there are sex differences in intentional learning over this age period, and (1b) whether sex differences differed in pre-teens (aged 8–10 years) versus young teens (aged 10–12 years). A second research question was whether (2a) there are differences between children of higher versus lower LPE families in intentional learning over the total age period, and (2b) whether LPE differences differed in pre-teens (aged 8–10 years) versus young teens (aged 10–12 years).

Sex differences and differences between children of higher and lower LPE families in intentional learning were investigated in two age groups because an increasing number of studies has repeatedly shown that the magnitude of sex differences on various cognitive abilities is influenced by age. For instance, results of the large-scale longitudinal study of Camarata and Woodcock (2006) showed that the effect on information processing speed was relatively small in young children (aged 9 years and younger), larger in early adolescence (aged 10–13 years), and the largest in middle adolescence (aged 14–18 years). In addition to sex, it could be that the magnitude of LPE differences also fluctuates with age. For instance, studies reported rapid brain maturation in childhood and early adolescence, followed by a more gradual rate, ultimately plateauing in early adulthood (e.g., Raznahan et al., 2011; Schnack et al., 2014). It is therefore possible that LPE especially influences brain maturation at early ages. Results of previous studies, thus, stress the need to examine the issue of sex and LPE differences in cognitive abilities during adolescence in narrow age classes. This is especially needed because when sex differences are investigated in group with broad age ranges, larger sex difference at particular ages will be reduced by the smaller differences at other ages, and average performance of boys and girls will be almost equal. The present study, therefore, investigates sex and LPE differences in intentional learning in two age groups with narrow age ranges.

## Materials and Methods

### Procedure and Participants

The data used in this study were derived from a large-scale cross-sectional study into the determinants of learning performance in students aged 8-12 years. All data were collected in April 2014 (van Tetering and Jolles, 2017). Participants were recruited from four regular mainstream primary schools in a rural area near the greater Amsterdam region (Netherlands). All schools belonged to one school organization with the same board involving 22 schools. The choice for the four schools was based on the presence of participants with a broad range of socioeconomic statuses (SES) ranging from low to high. The SES of the participants was evaluated according to the mean income and educational levels of the individuals living in the schools’ neighborhood (CBS Central Bureau for Statistics [CBS], 2016a,b). By including a roughly equivalent number of participants from low, moderate and high SES families we controlled for SES differences between participants to interfere with our main outcomes. Participation in the study was voluntary. All caregivers were informed that no personal information would be obtained and all data were assembled and analyzed at group level. The parents or caregivers (referred to as caregivers in the rest of this paper) gave written permission for their child to participate. The consent obtained from all caregivers of the non-adult participants was thus both informed and written. The Ethics Committee of the Faculty of Behavioral and Movement Sciences of the Vrije Universiteit Amsterdam approved the study protocol.

After permission of the caregivers for their child to participate, caregivers received an e-mail with login details for a short questionnaire which was presented via the internet. They were asked to indicate the highest level of education of both the father and the mother of the child. They indicated their level of education on a scale consisting out of 9 categories (0 = not finished any education to 9 = finished post university education). This classification is based on the International Standard Classification of Education (Singh, 2010).

Well-trained research assistants of the research center tested all children individually. Children were tested at their own school. A fixed battery with 8 neuropsychological tests was administered. Administration of the total battery took approximately 60 min. The second test within this battery was a measure of intentional learning.

#### Inclusion and Exclusion of Participants

In total, *N* = 310 subjects participated in the study. Data of participants were excluded from analysis when they accelerated or delayed a class (*n* = 81). Accordingly, the October-norm was used to create sharp age-boards between grades. The exclusion of participants was therefore based on their date of birth. An example in third grade: all participants born before 1 October 2004 or after 1 October 2005 were excluded (Rijksoverheid, 2017). This was done in order to have a relatively homogeneous sample with typically developing participants in each grade. School performance of all children was thus within the normal range and children with major learning disabilities that affected school performance were therefore excluded from the study sample. Participants were additionally excluded if data was unreliable due to technical problems (*n* = 3), or if data was missing on the LPE (*n* = 38). In addition, equal sex ratios between grades were created to control for sex effects within grades. Accordingly, boys and girls of the same school who had equal ages and were in the same grade were matched. Note that we reduced inter-group variance by matching girls and boys for each grade because we hypothesized that boys and girls differ in their intentional learning ability. Mean performance of one of the two age groups could be confounded by sex if there are unequal number of boys and girls in each age-group (mean performance of an age group will improve the more girls an age group contains). This procedure resulted in the exclusion of *n* = 30 boys and *n* = 6 girls (i.e., *n* = 7 boys in grade 3, *n* = 3 boys and *n* = 1 girl in grade 4, *n* = 12 boys and *n* = 2 girls in grade 5 and *n* = 8 boys and *n* = 3 girls in grade 6).

Data of *n* = 152 children were analyzed. These participants were divided in two age-groups, in which children within grades 3 and 4 belonged to age-group 1 (aged 8.6–10.7) and children within grades 5 and 6 (10.7–12.7 years) belonged to age-group 2 (see also earlier studies such as Levine et al., 1999; Camarata and Woodcock, 2006; Titze et al., 2010; Cross et al., 2011; Hoyek et al., 2011). This procedure has clinical and educational relevance because it enables to compare differences in the performance of younger and older children. Next to creating two age-groups, also LPE was dichotomized into two levels: low-to-moderate LPE (i.e., vocational training or lower, *N* = 67) and high LPE (higher than vocational training, *N* = 85). Dichotomization was based on the frequency distribution of LPE, so that two groups were created with comparable sample sizes. **Table 1** shows the demographic data for both age-groups. All children were Dutch speakers.

**Table 1:** Participants characteristics per age-group.

	Age group 1	Age group 2
*Group demographics*		
N	80	72
Age (mean (*SD*)^a^	9.6 (0.6)	11.5 (0.5)
Age-range (min-max)^a^	8.6–10.7	10.7–12.7
Sex distribution (N boys/N girls)	40/40	36/36
LPE (N low-moderate/N high)	36/44	31/41
Mean (*SD*) LPE^b^	6.4 (1.4)	6.5 (1.3)

*^a^Age in years; ^b^LPE was measured on a 9-point scale, ranging from 0 = no finished education to 9 = post university education.*

### Instrument: The Pictorial Verbal Learning Task

The multi-trial PVLT was used to measure intentional learning. Verbal learning tests are among the most often-used neuropsychological tests in applied settings and memory research, and the test-retest reliability has been reported to be high (Brand and Jolles, 1985; Van der Elst et al., 2005; Lezak et al., 2012). The PVLT consisted of three trials: each trial consisted of the presentation of the same 15 pictures of familiar objects. The words that the pictures referred to were controlled for frequency of use in the Dutch language (Linschoten, 1963) and the number of syllables and imageability (Van Loon-Vervoorn, 1989). The pictures refer to concrete objects such as hammer, vesta, beard and crane (see **Supplementary Figures 1**, **2**). Various possible categories of words (e.g., animals, body parts, parts of the house, furniture) were evenly distributed over the list in order to control for potential semantic and acoustic associations. Care was taken to avoid pictures with possible emotional connotations. The pictures were presented in the same order in each trial (Meijs et al., 2009, 2013, 2016).

Fifteen pictures were presented on the screen of a tablet (13.60 × 21.80 cm). The presentation duration was 1 s and there was an inter-stimulus interval of 1 s. The pictures (9.4 × 10.4 cm) consisted of line drawings and they were presented in black against a white background in the center of the screen. The distance between the participant and the tablet was approximately 30 cm. After each complete presentation, the participant had to verbally recall as many pictures as possible, regardless of the order that they were presented in. This procedure was repeated three times (trial 1–3). After a period of approximately 15–20 min, in which several information processing tests were administered that did not interfere with memory, a delayed recall trial was executed. The participant had to recall as many pictures as possible from the list without prompting (Meijs et al., 2009, 2013, 2016).

#### PVLT Outcome Measures

The following measures were analyzed: (1) Trial 1: the number of correctly recalled pictures after the first learning trial, (2) Trials 1–3: the total number of correctly recalled pictures over three learning trials, and (3) Delayed recall: the number of correctly recalled pictures after a 20-min delay in which the subjects engaged in simple information processing tasks. The immediate recall on trial 1 score is taken as an indication of a subject’s ability to deal with unfamiliar procedures and to learn newly presented information. The total number of pictures recalled, summed over the three learning trials reflects learning ability after repeated presentation of the same information. Lastly, the delayed recall is a measure of the ability to recall earlier learned information from long-term memory (Van der Elst et al., 2005).

#### Data Analysis

2 (age group: 1 vs. 2) × 2 (sex: boys vs. girls) Analyses of Variances (ANOVAs) on each of the PVLT outcomes (immediate recall trial 1, total recall trials 1–3, delayed recall) were conducted. Then, follow-up one-way ANOVAs were performed in each age group separately to assess whether the sex differences were present in both age groups. These analyses were performed because previous studies showed that the magnitude of sex differences in cognitive abilities can vary as a function of age (e.g., Camarata and Woodcock, 2006; Cross et al., 2011; see also Merrill et al., 2016).

The same analyses were performed to evaluate LPE differences in intentional learning: 2 (age-group: 1 vs. 2) × 2 (LPE: moderate-to-low vs. high) ANOVAs on each of the PVLT outcomes (immediate recall trial 1, total recall trials 1–3, delayed recall) were conducted. Then, follow-up one-way ANOVAs were performed in each age group separately to assess whether the LPE differences were present in both age groups (for procedure see Merrill et al., 2016).

The assumptions for homogeneity of covariance matrices (i.e., Levine’s test) and normality were approved (i.e., visual inspection of the histograms and the normal probability plots, and skewness < 3, kurtosis < 10; Kline, 2005). A *p*-value < 0.05 was considered statistically significant. Partial eta’s squared were reported for significant findings as a measure for effect sizes. All analyses were performed using SPSS version 23.

## Results

### Sex Differences, Age and PVLT Performance

The main effects of age group on Trial 1 [*F*(1,148) = 8.32, *p* < 0.01, ${\text{η}}_{\text{p}}^{2}$ = 0.05], Trials 1–3 [*F*(1,148) = 13.33, *p* < 0.01, ${\text{η}}_{\text{p}}^{2}$ = 0.08] and delayed recall [*F*(1,148) = 14.46, *p* < 0.01, ${\text{η}}_{\text{p}}^{2}$ = 0.09] were significant, with the older group demonstrating better performance than the younger group.

The main effect of sex on Trial 1 [*F*(1,148) = 5.13, *p* < 0.03, ${\text{η}}_{\text{p}}^{2}$ = 0.03] and on Trials 1–3 [*F*(1,148) = 3.99, *p* < 0.05, ${\text{η}}_{\text{p}}^{2}$ = 0.03] were also significant. On both outcome measures, girls demonstrated better performance than boys. The main effect of sex on delayed recall was not significant [*F*(1,148) = 2.14, *p* = 0.15]; the same applied to the interaction effects between age group and sex on any of the PVLT outcomes [Trial 1: *F*(1,148) = 2.52, *p* = 0.11; Trials 1–3: *F*(1,148) = 2.20, *p* = 0.14; delayed recall: *F*(1,148) = 0.41, *p* = 0.52].

The follow-up analyses that were performed to compare the performance of girls and boys in each age group revealed a significant difference between boys and girls in the older group on Trial 1 [*F*(1,71) = 7.60, *p* < 0.01, ${\text{η}}_{\text{p}}^{2}$ = 0.1] and on Trials 1–3 [*F*(1,71) = 5.71, *p* = 0.02, ${\text{η}}_{\text{p}}^{2}$ = 0.08]. Girls performed better than boys. Means and standard errors for each age group and for boys and girls on the three PVLT outcome measures are presented in **Table 2**. Results of the *post hoc* analyses are presented in **Table 3**.

**Table 2:** Mean and Standard Error per Age-group, Sex and Level of Parental Education for each PVLT outcome measure.

*PVLT outcomes*						
	**Trial 1**		**Trial 1–3**		**Delayed recall**	
	**Mean**	***SE***	**Mean**	***SE***	**Mean**	***SE***
Age-group 1 (8–9.9 years)	7.1	0.21	27.9	0.50	9.8	0.22
Age-group 2 (10–12.9 years)	7.9	0.22	30.5	0.55	10.9	0.21
Boys	7.1	0.22	28.4	0.55	10.1	0.23
Girls	7.8	0.21	29.8	0.52	10.5	0.21
Low-to moderate LPE	6.9	0.23	28.1	0.58	10.0	0.24
High LPE	7.9	0.20	29.9	0.49	10.6	0.21

**Table 3:** Sex differences on PVLT-performances per age-group.

Age group 1						Age group 2				
	Boys		Girls			Boys		Girls		
	Mean	*SE*	Mean	*SE*	*P*-value	Mean	*SE*	Mean	*SE*	*p*-value
Trial 1	7.0	0.31	7.2	0.28	0.64	7.3	0.31	8.5	0.27	<0.01^∗^
Trial 1–3	27.7	0.72	28.1	0.69	0.71	29.3	0.84	31.8	0.64	0.02^∗^
Delayed recall	9.7	0.33	9.9	0.28	0.57	10.6	0.32	11.3	0.28	0.14

*^∗^p < 0.05.*

These results indicate that older subjects performed better than younger participants on immediate recall after trial 1, total trials 1–3 and on the delayed recall of the PVLT. In addition, sex differences in performance were present in the older subjects but not in the younger group, with girls outperforming boys.

### LPE, Age and PVLT Performance

The main effects of age on Trial 1 [*F*(1,148) = 8.09, *p* < 0.01, ${\text{η}}_{\text{p}}^{2}$ = 0.05], Trials 1–3 [*F*(1,148) = 12.38, *p* < 0.01, ${\text{η}}_{\text{p}}^{2}$ = 0.08] and delayed recall [*F*(1,148) = 13.335, *p* < 0.01, ${\text{η}}_{\text{p}}^{2}$ = 0.08] were significant. The older group performed better than the younger group.

The main effect of LPE was significant on Trial 1 [*F*(1,148) = 9.24, *p* < 0.01, ${\text{η}}_{\text{p}}^{2}$ = 0.06] and on Trials 1–3 [*F*(1,148) = 5.94, *p* < 0.02, ${\text{η}}_{\text{p}}^{2}$ = 0.04]. The main effect of LPE on the delayed recall approached significance [*F*(1,148) = 3.50, *p* = 0.06, ${\text{η}}_{\text{p}}^{2}$ = 0.02]. The high LPE group performed better than the low-to-moderate LPE group. No significant interactions between age group and LPE were found on any of the PVLT outcomes [Trial 1: *F*(1,148) = 0.03, *p* = 0.87; Trials 1–3: *F*(1,148) = 0.62, *p* = 0.62; delayed recall: *F*(1,148) = 0.80, *p* = 0.37]. Follow-up analyses comparing the performance of participants of high and low-to-moderate LPE families in each age group revealed a significant difference in mean performance on Trial 1 in the younger group [*F*(1,79) = 5.40, *p* = 0.02, ${\text{η}}_{\text{p}}^{2}$ = 0.07]. In the older group, the differences in performance on Trial 1 [*F*(1,71) = 3.95, *p* = 0.05, ${\text{η}}_{\text{p}}^{2}$ = 0.05], Trials 1–3 [*F*(1,71) = 4.00, *p* = 0.05, ${\text{η}}_{\text{p}}^{2}$ = 0.05] and on the delayed recall [*F*(1,71) = 3.97, *p* = 0.05, ${\text{η}}_{\text{p}}^{2}$ = 0.05] were significant. Mean performance of the high LPE group was better than the mean performance of the low-to-moderate LPE group. Means and standard errors for each age group and for the low-to-moderate and high LPE groups on the three PVLT outcome measures are presented in **Table 2**. **Table 4** presents the means and standard errors of both LPE groups per age group.

**Table 4:** Level of parental education differences on PVLT-performances per age-group.

Age group 1						Age group 2				
LPE										

	Moderate-to-low		High			Moderate-to-low		High		
	Mean	*SE*	Mean	*SE*	*p*-value	Mean	*SE*	Mean	*SE*	*p*-value
Trial 1	6.5	0.29	7.5	0.28	0.02^∗^	7.4	0.36	8.3	0.26	0.05^∗^
Trial 1–3	27.1	0.75	28.5	0.66	0.16	29.3	0.88	31.4	0.66	0.05^∗^
Delayed	9.6	0.36	9.9	0.26	0.50	10.5	0.31	11.3	0.28	0.05^∗^
Recall										

^∗^*p < 0.05.*

Taken together, a significant difference between the low-to-moderate and high LPE group was found on immediate recall after trial 1 in the younger age group. In the older age group, significant differences in performance between the high and low-to-moderate LPE group were found in immediate recall after trial 1, total recall trials 1–3 and the delayed recall.

### *Post hoc* Power Analyses

*Post hoc* power analyses were performed because the results revealed no significant interaction effects between sex and age group and between LPE and age group, which contrasts the findings of our follow-up analyses. These findings showed sex and LPE differences in one of the separate age groups, but not necessarily in the other. We therefore performed *post hoc* power analyses to calculate the required sample size to detect a significant interaction effect. Calculations (using G-power 3.1) revealed that our sample size was too small to detect a moderate significant interaction effect (${\text{η}}_{\text{p}}^{2}$ = 0.16, i.e., required sample size was 523).

## Discussion

This study investigated whether the child’s sex and LPE contributed to individual differences in intentional learning in students aged 8–12 years. Intentional learning was administered with the aid of a newly constructed multi-trial learning task; the PVLT. This task evaluates intentional learning of visually presented pictorial material (line drawings of common animated and unanimated objects and material). Our results revealed that boys and girls differed in PVLT performances. These sex differences were confined to the older age group (i.e., 10.7–12.7 years): Older girls recalled more pictures than older boys after the first presentation of pictures (i.e., Trial 1) and in total (i.e., the number of recalled pictures summed over the three learning trials). This is an important new finding because it has repeatedly been documented that boys have more experience with the processing of complex visual information than girls (e.g., Miller and Halpern, 2014). These experiences are the consequence of their previous learning experiences and preferences: Boys have a preference for construction materials, whereby they gain experience with building towers out of blocks that are presented on pictures (see also the review of Miller and Halpern, 2014). These previous experiences with processing visual information could have been advantageous for PVLT-performance. Our results showed that despite the superior spatial skills of boys, girls outperform boys on tasks that evaluate the ability to learn pictorial information. This points to a possible involvement of other important factors such as a higher motivation to learn and the use of better learning strategies in girls. In addition to sex differences, results also revealed LPE differences in PVLT performance in both younger and older children. Children from higher LPE families recalled significantly more pictures directly after the first presentation of pictures. Sex and LPE differences can thus be considered as two potent factors contributing to individual differences in intentional learning performance in schoolchildren.

### Sex Differences in Intentional Learning

Our results suggest that the development of intentional learning in boys is lagging behind that of girls at approximately 10–12 years of age. We performed *post hoc* analyses to investigate whether the difference in performance after the repeated presentation of information (i.e., Trials 1–3) was the result of the difference in performance after the first presentation of information (i.e., Trial 1). Results showed that this indeed was the case: The shortfall in performance after the presentation of repeated information of boys was due to the difference in performance after the first presentation of information. This result indicates that at the age of 10–12 years, boys have more difficulties with the initial encoding of new procedures and unfamiliar information than girls. This finding is of applied value for education as it suggests that boys need more guidance than girls when a new task is introduced.

Our finding is supported by earlier studies which showed that the magnitude of sex differences in cognitive abilities varies as a function of age (Camarata and Woodcock, 2006; Cross et al., 2011). Sex differences in intentional learning may only be present after the age of 10 years. Our findings therefore highlight the importance of investigating sex differences in intentional learning and other cognitive abilities in populations with narrow age-ranges.

The fact that the current study revealed sex differences in older, and not in younger children may have to do with boy-girl differences in the ability to process complex visually presented information. Quite a few studies have shown that (the majority of) boys are somewhat better than (the majority of) girls in this domain (e.g., Voyer et al., 1995; Hoyek et al., 2011; Miller and Halpern, 2014). This may have given the young boys some advantage when processing pictorial information presented as to-be-learned information as on the PVLT (Miller and Halpern, 2014). At later ages however, girls may have developed significantly higher learning motivation (Dekker et al., 2013a) and better executive functions than boys (Hyde and Linn, 1988; Hyde, 2014; van Tetering and Jolles, 2017). As a result, girls could be better than boys in concentrating on tasks as well as on procedural learning, and the better visuospatial processing skills of boys cannot compensate anymore.

Sex differences in various cognitive abilities may thus contribute to the sex difference in intentional learning performance as reported in our study (Diamond, 2013; Baars et al., 2015). For instance, previous studies reported lower levels of attention for boys compared to girls (e.g., van Tetering and Jolles, 2017). Boys may therefore have more difficulties with focusing their attention while performing the PVLT than girls, which could negatively affect task performance. The sex difference in PVLT performance may further be explained by differences in motivational factors between boys and girls. During adolescence, large developments take place in a student’s beliefs and academic self-perceptions, such as their perceived competence and the value they place on doing well (see, for instance, Bouchey and Harter, 2005). Adolescent boys were found to have less adaptive school motivation than girls. This could possibly explain their lower achievement in school-related tasks (Van Houtte, 2004; Dekker et al., 2013a), as well as on the PVLT. Overall, boy–girl differences in various cognitive abilities can contribute to the sex differences in intentional learning performance as reported in our study. Future research should therefore investigate the importance of various cognitive abilities to intentional learning performance. The existence of sex differences in the cognitive abilities that are important for intentional learning performance could enable the formulation of learning methods and procedures to become more beneficial in learning because previous learning methods can be adjusted to the abilities of boys and girls. For instance, it could be that boys need additional explanation when tasks are presented for the first time because they have lower levels of sustained attention than girls. Adjusting previous learning methods and procedures in a way that boys get additional explanations could be beneficial for the learning outcomes of boys.

### LPE Differences in Intentional Learning

In addition to sex differences, we found that children of more highly educated parents outperformed children of parents that attained lower educational levels after the first presentation of information (i.e., Trial 1). This finding suggests that children of more highly educated parents may be better at processing new information and unfamiliar procedures. In the older group, results revealed that children from higher LPE families also outperformed children from lower LPE families after the repeated presentation of the same information (i.e., total recall summed over three learning trials). Nevertheless, the *post hoc* analyses revealed that this difference in performance after the repeated presentation of information was the result of the difference in performance after the first presentation of information. This finding is important since previous studies reported that there are differences in the cognitive abilities of children from lower and higher LPE families that could contribute to individual differences in school achievement (Ganzach, 2000; Evans et al., 2010; Carr and Pike, 2012; Rindermann and Baumeister, 2015). The results of our study more specifically suggest that these children differ in their ability to process unfamiliar procedures and information, and not in their learning abilities *per se*.

It has often been suggested that the better cognitive performance of children from higher LPE families is related to a better genetic predisposition for learning (see van Soelen et al., 2009; Sameroff, 2010; Trzaskowski et al., 2014). Another possibility is that the difference results from environmental factors such as a more inspiring and intellectually stimulating atmosphere of the family in which children grow up. It appears that higher education of parents is the driver of that advantage since previous studies reported relations between LPE and the motivational encouragement and intellectual (verbal) stimulation by parents (Ganzach, 2000; Evans et al., 2010; Carr and Pike, 2012; Rindermann and Baumeister, 2015). This may encourage their children to gain more experience in playing with new and unfamiliar games or with reading new books (Rindermann and Baumeister, 2015). Experiences with new materials are relevant, for example, when children have to take a test at school in which the procedures are unfamiliar. Our findings, therefore, suggest that it is important to support parents with lower LPE and guide them to present their children with new learning materials and to stimulate their insights and knowledge about how to stimulate the cognitive development of their children. This could provide their children with the opportunity to gain experience in processing new information and procedures and to develop better learning strategies accordingly.

From a neuropsychological perspective, we investigated LPE differences on separate, distinctive cognitive abilities which were administered using one task. It was expected that LPE could selectively affect some of the outcome measures and not others. It is of special relevance for future research to replicate our findings in a larger study to determine whether the effects of LPE that were found in this study remain significant. Future research should use a more specific measure of LPE. LPE was dichotomized in our study which was the best option given the sample size. Nevertheless, there are considerable differences in the degree to which caregivers create an intellectually stimulating learning environment for their children within one of the two LPE groups of our study (e.g., between the lowest educated parents and those who obtained moderate educational levels). Also, a more specific measure of LPE should be devised in order to incorporate additional educational credits which the caregivers may have obtained (e.g., in corporate training and post-initial education). Accordingly, future studies should take the current professions of caregivers into account to investigate the importance of past education for intentional learning.

In order to interpret the results presented here correctly, we address three points that are important to be taken into consideration in future studies and in educational practice. First, we took advantage of our large dataset. The use of such a large group has a major advantage of allowing controls for interferences from external variables with our outcome measure, namely: (a) the study was performed at four primary schools with the same educational board to reduce possible differences in background related to the regional geography or in the educational philosophy of the schools; (b) during the selection of primary schools, the SES background of participants was taken into account in order to include a broad spectrum of students with low to high SES families; (c) the sample was homogenized with respect to confounding variables, such as repeating or skipping a grade, to reduce variance within grades due to age differences (e.g., older children may have advanced cognitive development compared to younger children). This procedure resulted in the inclusion of children with school performances within the normal range (children with dyslexia that had normal school achievement were included in our study sample). Note that our results are therefore not generalizable to individuals with severe learning disabilities. Another advantage of our large study sample is that (e) we distributed boys and girls equally among the grades to make sure that the better performance in one of the grades was not caused by differences in the boy–girl distribution since our hypothesis was that girls outperformed boys. Moreover, (f) we performed *post hoc* analyses to make sure that children of the four schools were equally distributed over the sexes, LPE groups, and age groups; and (g) to investigate whether the unequal number of participants in the moderate-to-low and high LPE groups affected our results. Results of these *post hoc* analyses were essentially the same (results not published). This strict stratification allowed us to focus evaluation on the core factors, LPE and sex, without external factors interfering.

The second point that needs attention is the size of the significant difference in mean performance on the PVLT between boys and girls, and between children from higher and lower LPE families. The significant differences in mean performances on Trial 1 of boys and girls is around 0.7 words, and between high and moderate-to-low LPE is around 0.8 words. As we see in our results, the standard errors are larger for boys than girls, and for the group of children with moderate-to-low LPE compared to high LPE. This indicates that the lowest performing boys perform even worse than the lowest performing girls. The same accounts for LPE: The lowest performers in the moderate-to-low LPE group performed even worse than the lowest performers in the higher LPE group. This is an indicator that there are quite a number of substantial individual differences within the groups. The small difference in mean performance could be explained by the fact that there could be boys that perform equally as well as girls, and children with low-to-moderate LPE that perform equally to children with high LPE, even while the variation on PVLT performances is much greater in boys than in girls, and in children with low-to-moderate LPE compared to children with high LPE.

A final note that needs to be addressed is the fact that results revealed no significant interaction effects between sex and age group and between LPE and age group. This is in contrast with the findings of our follow-up analyses. These findings showed sex and LPE differences in one of the separate age groups, but not necessarily in the other. The *post hoc* findings showed that a two-way ANOVA requires more power than our present sample with 152 subjects enables. It is therefore needed to re-investigate our findings in a future study with a larger sample size. At this moment, the findings from our study are straightforward in that boy–girl differences and LPE differences are dependent upon age.

### Implications and Conclusion

In this study, the PVLT has been used to assess intentional learning performance (Meijs et al., 2009, 2013, 2016). This assessment tool is easy to use in applied settings such as school or in clinical settings. The PVLT is a multi-trial list-learning task in which pictures (line drawings) should be remembered. A major advantage of the PVLT is that it assesses gnostic information processing which requires the subject to recall a visual presentation of objects or materials without the interference of reading difficulties. This is an advantage since there is evidence that during late childhood and young adolescence, many children experience some problems in learning to read, and there are major differences in the pace at which reading skills develop between children (e.g., Cecilia et al., 2014). The PVLT could, therefore, be more useful than traditional learning tests (e.g., AVLT) (Rey, 1964) to evaluate intentional learning performance in children who lag somewhat behind in normal development of reading abilities as well as in children with hearing difficulties. As found in the present study, the PVLT appears to be a relevant instrument which is applicable in a school context and in clinical settings.

## Conclusion

This study offers important new insights into factors which can contribute to individual differences in intentional learning in the transition period from childhood to adolescence. Intentional learning is a major aspect of learning at school and in daily life outside of school. The findings of the present study indicate that the sex of the child and the learning environment, as created by parents and school, are factors which can be important determinants to the development of individual differences in intentional learning. This study contributes to a better understanding of differences and similarities in the neuropsychological development of boys and girls and between children growing up in lower and higher LPE families. These are insights which – from the perspective of applied neuropsychology – can have important consequences for educational innovations and improvement of learning performance at school (see Camarata and Woodcock, 2006). They allow the adjustment of learning materials and procedures to each students’ needs in order to optimize learning outcomes. In fact, the findings suggest that children of less educated parents and boys should be given special attention and guidance in situations which require intentional learning, especially when new procedures and tasks are presented for the first time.

## Author Contributions

MvT: substantial contributions to the conception and design of the work, the analysis, and interpretation of data for the work, drafting the work, final approval of the version to be published, and agreement to be accountable for all aspects of the work in ensuring that questions related to the accuracy or integrity of any part of the work are appropriately investigated and resolved. RdG: substantial contributions to the design of the work, the interpretation of data for the work, revising the work critically for important intellectual content, final approval of the version to be published, and agreement to be accountable for all aspects of the work in ensuring that questions related to the accuracy or integrity of any part of the work are appropriately investigated and resolved. JJ: substantial contributions to the conception or design of the work, the acquisition and interpretation of data for the work, revising the work critically for important intellectual content, final approval of the version to be published, agreement to be accountable for all aspects of the work in ensuring that questions related to the accuracy or integrity of any part of the work are appropriately investigated and resolved.

## Conflict of Interest Statement

The authors declare that the research was conducted in the absence of any commercial or financial relationships that could be construed as a potential conflict of interest.
