# Supplementary material for: Boy–Girl Differences in Pictorial Verbal Learning in Students Aged 8–12 Years and the Influence of Parental Education
Source: Front Psychol. 2018 Aug 8;9:1380. doi: 10.3389/fpsyg.2018.01380 (PMC6092633; doi:10.3389/fpsyg.2018.01380)
Supplement: Supplementary file 1 [file Image_1.PDF]

Supplementary Material

Boy-Girl differences in Pictorial Verbal Learning in Students Aged 8–12 Years and the Influence of Parental education

M.A.J. van Tetering[1*], R. de Groot[2,3], J. Jolles[1]

*** Correspondence:** M.A.J. van Tetering: m.a.j.van.tetering@vu.nl

1 Supplementary Figures

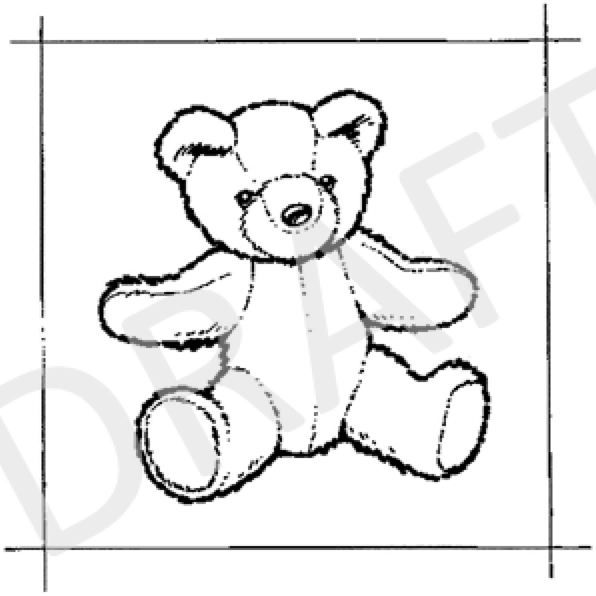

DRAFT

Supplementary Figure 1. Example of a picture used in the Pictorial Verbal Learning Test
